# Post Hospital Discharge Functional Recovery of Critical Illness Survivors. Systematic Review

**DOI:** 10.2478/jccm-2023-0011

**Published:** 2023-05-08

**Authors:** Irini Patsaki, Georgia Bachou, Georgios Sidiras, Serafim Nanas, Christina Routsi, Eleftherios Karatzanos

**Affiliations:** 1 University of West Attica, Athens, Greece; 2 National and Kapodistrian University of Athens, Athens, Greece; 3 Clinical Ergospirometry, Exercise and Rehabilitation Laboratory, 1st Critical Care Department, Evangelismos Hospital, School of Medicine, National & Kapodistrian University of Athens, Athens, Greece

**Keywords:** physical function, functional rehabilitation, quality of life of ICU survivors, critically illness

## Abstract

**Background:**

Millions of people face critical illnesses and need to be hospitalized in an Intensive Care Unit (ICU) annually worldwide. Despite the fact that survival rates of these patients have increased, they develop various cognitive, psychological and functional impairments. This study aims to investigate the significance of the recovery interventions following intensive care unit discharge, the effectiveness of the rehabilitative protocols and their possible deficits.

**Methods:**

MEDLINE (PubMed) and Physiotherapy Evidence Database (PEDro) were searched for studies analyzing the recovery potentials post-ICU among adults, who spent at least 48 hours at the ICU. Methodological quality of the studies was assessed via PEDro Scale.

**Results:**

Nine randomized controlled trials were included. These took place mainly at specialized rehabilitation gyms as well as patients home environments. Studies analyses showed that treatment group showed improvement in functional ability in relation to control group. Nevertheless, differences between two groups were not statistically significant (P<0.05). The majority of studies assessed cardiorespiratory endurance and muscular strength.

**Conclusions:**

The included rehabilitation programs were determined to be effective. Although they didn’t prove any statistically significant difference between groups, quality of life enhancements and stress reduction were reported. Hence, new randomized controlled trials are required in order to provide more accurate data on the potential benefits of rehabilitation strategies among post-ICU patients.

## INTRODUCTION

Advances in critical care medicine and technological breakthroughs have led to a significant increase in critical illness survivorship. Taking into consideration the increasing number of patients that are in need of Intensive Care Support especially nowadays that humanity has been faced with a pandemic, special attention should be given to survivors and their rehabilitational needs. It’s being widely supported that survivors of critical illness face serious deficiencies that affect their quality of life years after their hospital discharge [1–3]. Post-Intensive Care Unit patients suffer from a variety of physical, cognitive and psychological problems. Patients suffer from significant muscle weakness [2], dysphagia [4], persistent pain[5], anxiety, depression and even memory deficiencies [6]. These being manifested alone or in a combination, have led the medical community to enclose them under the term “Post Intensive Care Syndrome-PICS” [7]. For the best resolution of these problems, survivors of critical illness are often led to in-patient or out-patient rehabilitation facilities. The impact of PICS in patients’ functional ability and quality of life is enormous and has significant extensions to health care systems and the country’s economy. Different rehabilitation programs and innovative equipment such as neuromuscular stimulation [8] or even virtual reality [9] have been used in an effort to enhance and speed up recovery process. Rehabilitation having started within the ICU through early mobility strategies continues after ICU and hospital discharge. Multicomponent rehabilitation programs have been suggested to be most suitable to address the diversity of PICS, yet there is still little evidence to support the most effective program. The combination pf physical, mental and psychological impairments is a challenge for the rehabilitation team. Post -ICU recovery remains a mystery and a controversial theme especially after hospital discharge as a percentage of survivors will continue to struggle to return to normal leaving. The purpose of this systematic review was to investigate the effectiveness of rehabilitation interventions in post ICU survivors after their hospital discharge in relation to their functional ability.

## METHODS

### Search Criteria and study identification

We systematically searched Medline (Pubmed) and Physiotherapy Evidence Database (PEDro) using search strategies based on keywords such as physical function, physical recovery, functional ability, functional rehabilitation, exercise, post ICU, quality of life, ICU survivors, post critical illness, hospital discharge. Search strategies developed combining the terms mentioned, for example: “post ICU OR ICU survivors” and “physical recovery OR functional rehabilitation”. Additionally, hand search was performed in all the references list of the articles that were identified. The databases were screened for articles published from January 2010 to November 2021 from two independent experienced clinicians.

The Inclusion criteria were:

– Study design: all randomized controlled trials
– Study population: critically ill adults that were admitted in the Intensive Care Unit (ICU) for at least 4 days and being under mechanical ventilation ≥48 hours.
– Intervention: physical or functional rehabilitation program after hospital discharge
– Outcomes: physical or functional status, activities of daily living, muscle strength

The exclusion criteria were:

– Pediatric population
– Animal studies
– Being under mechanical ventilation <48hours
– Interventions being implemented from ICU stay
– Published in non-English Language
– No physical rehabilitation intervention, for example only cognitive one

Two reviewers independently screened the titles and abstracts for relevant studies and the same reviewers assessed the full text of all eligible studies and potential disagreements were discussed and resolved with a third author.

### Methodological quality

The quality of the included studies was evaluated by the Physiotherapy Evidence Database (PEDro) Scale. Total scores from 6 to 10 considered high quality, from 4-5 considered fair quality and ≤ 3 considered poor quality. Two authors conducted a blinded rating of the methodological quality of the studies. Different rates and unclear issues were discussed, and disagreements were resolved with a third author.

## RESULTS

### Study selection and Quality

**Fig. 1.**
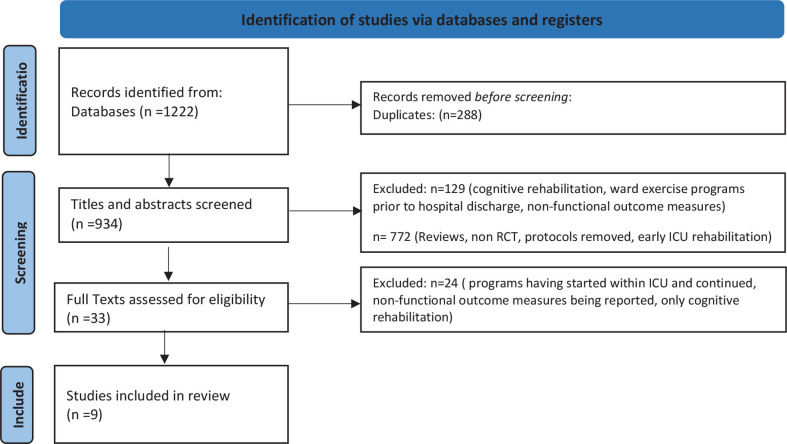
Prisma Flow diagram

A total of 1222 studies were identified from the search and after removing duplicates 934 studies were screened based on their title and abstract. Thirty-three full text articles were assessed based on the inclusion and exclusion criteria of the review. Twelve studies were excluded as rehabilitation was initiated from the ICU, 4 studies didn’t assess functional or physical performance and another 8 involved only cognitive rehabilitation and all were excluded as well. Finally, only 9 studies[10–18] fulfilled the criteria (Table 1) and were included in this systematic review (Figure 1).

**PRISMA 2020 flow diagram for new systematic reviews which included searches of databases and registers only**

**Table 1.** Description of the studies included in the systematic-review.

Study	Group (N) APACHE-II	Intervention	Configuration	Duration	Outcomes
Batterham et al. (2014)	CG:30 APACHE-II:16.4(7.8) IG:29 APACHE-II:15.9(7.9)	CG: Standard care(medical follow-up, no rehabilitation) IG : Program with cycle ergometer 30′ of moderate intensity, 2 supervised sessions/1 unsupervised	SF-36 (PF)	9 weeks Follow up: 26 weeks	SF-36 (PF): IG : 43.5 (18)vs CG: 40.1(23), 95% confidence interval [-1.4-8.2])
Battle et al. (2018)	CG :30 APACHE-II:15 (12-19) IG :30 APACHE-II:13 (9-19)	CG : Standard care (no rehabilitation prescription) IG : Personalized supervised exercise program in an outpatient gym	6MWT, Berg balance, HG	6 weeks after hospital Follow up:7 weeks, 6 months, 12 months	At 6 weeks 6MWT: IG:379.3(207.9) vs CG:283.6(229.3), p=0.491 Berg :IG :53.4(6.2) vs CG:50.5 (7.0), p=0.99 HG(L): IG:20.7(11.4) vs19.5(12.5),p=0.287
Connolly et al. (2015)	CG :10 APACHE-II:23.5 (21-30.3) IG :10 APACHE-II:24.5(18.8-29.5)	CG: Weekly telephone follow-up IG: 16 sessions in an outpatient gym	ISWT, 6MWT, SF-36 (PCS),	3 months after hospital	Change in outcomes ISWT(m): CG: 170(40-315)vs IG: 115(-2.5-237.5) 6MWT(m):CG:185(40-285)vs IG:140(35.8-210.3) PCS: CG: 11(4.3-28.3) vs IG:1.8 (-6.8-15.9)
Elliot et al. (2011)	CG: 91 APACHE-II:19.5(7.2) IG: 92 APACHE-II:19.4(12.6)	CG : Standard care (medical follow-up) IG : Home rehabilitation program with an emphasis on increasing muscle strength & walking	SF-36 (PF), 6MWT	8 weeks Follow up: 26 weeks	SF-36 (PF) :IG :39.9 vs CG :41.0, p<0.05, while the effect time was notable for PF (p=0,034) and for 6MWT(p=0,0003).
McDowell et al. (2016)	CG : 30 APACHE-II:15.2(5.6) IG : 30 APACHE-II:17.3 (7.7)	CG : Standard care(no support) IG : personalized exercise program in the hospital gym (supervised-2 sessions) or at home (unsupervised-1 session)	SF-36(PF),ISWT HG	6 weeks Follow up: 6 months	SF-36(PF) :IG :6.8(10.9) vs CG:3.9(8.2),p=0,26 ISWT : IG :135.5(119.8) vs CG:52.4(126.7),p=0.03 HG(dominant): IG:6.1(28.1)vs CG:12.5(22.8), p=0.39
McWilliams et al. (2016)	CG :36 APACHE-II:15.9(5.3) IG :37 APACHE-II:16.6(5.7)	CG : Standard care (no exercise prescription or consultation) IG : supervised session with a 10-station program-cardiovascular exercises	SF-36 (PCS),	7 weeks	Degree of improvement SF-36 (PF) IG :3.5(95% CI;1.6-6.7 vs CG :8.6(95%;5.4-10.6), p=0,048)
Vitacca et al. (2016)	CG :24 IG :24	OE: Standard care (medical support, free to conduct physical activity without monitoring or support) IG : pulmonary rehabilitation program at home under supervision	MIP/MEP, BADL, MRC-SS,	6 months	MIP: IG : +33cm H2O vs CG : +26cm H2O,p<0.03 MEP :IG : +42cm H2O vs CG : +28cm H2O, p<0.03 BADL: p=0.63 Biceps MRC-SS: p=0.07 Quadriceps MRC-ss:p=0.53
Veldema et al. (2019)	CG : 14 IG : a)13 b)12	OE: Standard care IG : a) cycle ergometer program, b) resistance program (inpatients in a neurological rehabilitation clinic) 5times/week, 4 sets of 15 repititions	FAC, TUG test, 10MWT, MMS test SF-36(PF)	2 weeks 4 weeks	6MWT (4 weeks): p<0.05 within groups TUG(4weeks):p<0.05 within groups FAC(2weeks):p<0.05 between groups MRC knee flexion (2weeks): p<0.05 between groups
Shelly et al (2017)	CG:18, IG:17	IG: 4 weeks unsupervised home rehabilitation program CG: no exercise prescrition	SF-36 (PF)	4weeks	SF-36 (PF ): IG: 10.32(8.51-14.92) vs CG: 7.36(3.68-8.48), p=0.003

IG: Intervention Group, CG: Control Group, 6MWT: 6 Minute Walk Test, ISWT: Incremental Shuttle Walk Test, PF: Physical Functioning, PCS: Physical Component Summary, TUG: Time Up and Go,STS-5: Sit-to-Stand 5times, HG: Hand Grip, ICUaw: Intensive Care Unit Acquired weaknsess, FAC: Functional Ambulation Category test, MMS: Manual Muscle Test, MRC-ss: Medical Research Council-strength scale, BADL: Basic Activities of daily living, MIP: Maximal Inspiratory Pressure, MEP: Maximal Expiratory Pressure Values are represented either as Median(IQR) or Mean (SD)

All studies included were assessed using the PEDro scale (Table 2). According to that 4studies [10–13] were considered as “high quality” and another 5 [14–18] as “fair quality”. The mean score of the methodological quality for the included studies was 6.2.

**Table 2.** Quality of the RCT studies of stroke patients on Pedro Scale (item1 doesn’t contribute to total score)

	Batterham et al (2014)	Battle et al (2018)	Connolly et al (2015)	Elliott et al (2011)	McDowell et al (2016)	McWilliams et al (2016)	Vitacca et al (2016)	Veldema et al (2019)	Shelly et al (2017)
1	√	√	√	√	√	√	√	√	√
2		√	√	√	√	√	√	√	√
3	√	√		√	√				√
4		√		√	√	√			√
5				√					
6	√						√		
7	√	√			√	√		√	
8				√	√	√		√	
9									
10	√	√	√	√	√	√	√	√	√
11	√	√	√	√	√	√	√	√	√
**Total**	**6/10**	**7/10**	**4/10**	**8/10**	**8/10**	**7/10**	**5/10**	**6/10**	**5/10**

### Participants

The studies included 565 participants (intervention group: 282 and control group: 283). The sample size ranged from 20 to 183 participants. Of the 530 patients, approximately 25% were respiratory patients, 18% were cardiac, and a small percentage of 3% were neurological patients. There are two studies that recruited only patients with ICU-acquired weakness [14,17]. The remaining patients had other pathological conditions (such as sepsis) and weren’t categorized so that they can be accounted. Most of the studies had a drop-out rate around 15-25%. Only the study of Battle et al. reached a quite higher percentage of patients (43%) that were lost to follow up. Participants were either recruited once being discharged from hospital or a few weeks after [11–13,15]. There were two studies that mentioned recruitment to take place even 3-4 months post hospital discharge [12,15].

### Rehabilitation strategies

Most studies were contacted in outpatient rehabilitation facilities [11–14,15,17], one was performed at inpatient neurological rehabilitation hospital [16], and four other were home based. In most studies, cardiopulmonary rehabilitation programs were performed using a cycloergometer, walking treadmill and rowing machines [10–12, 14–17] (Table 3). Six studies included additional interventions such as upper and lower limb and torso strengthening exercises, balance exercises, breathing exercises, functional exercises, flexibility exercises and application of electro-neuromuscular stimulation to the lower extremities [10–12, 14, 16,17]. Shelly et al investigated the effectiveness of an individualized home program of different strengthening and breathing exercises that patients were instructed to perform on their own. Several programs included warm-up and recovery. The total duration ranged from 20 to 90 minutes in three studies [11,16,17], whilst in other six up to 30 minutes [10,13–15,18]. The frequency varied from 2days/week [12,15,17], up to five days/ week [10,14,18] or even six [16]. The intensity of the exercise was gradually increasing and specifically in 5 studies out of 8 it was moderate to severe [10,11,13–15]. Program being designed for home rehabilitation were either totally unsupervised [10,18] or included both supervised and unsupervised sessions [11,13,15]. Studies that included unsupervised sessions did underline their inability to monitor patients’ adherence to the rehabilitation program.

**Table 3.** Description of the rehabilitation program that the intervention group followed.

Study	Duration / Intensity / Frequency	Intervention
**Batterham et al. (2014)**	Duration: 30 minutes	Cardiorespiratory exercises: cycle ergometer (12-14 Borg Scale)
	Intensity: moderate and progressively increasing	3 minutes at 60rpm and 3 minutes break
	Frequency: 2 times/week	Pedal resistance increased by 10 and 20W/min (The program included warm-up and recovery)
**Battle et al. (2018)**	Duration: 60 minutes	Cardiorespiratory exercises: cycle ergometer, treadmill, rowing machines
	Intensity: progressively increasing	Muscle strength and balance exercises with strength balls and bands, weights
	Frequency: 2 times/week	Functional balance exercises in a sitting and standing position, balance board
**Connolly et al. (2015)**	Duration: 40 minutes	Cardiorespiratory exercises
	Intensity: progressively increasing	Exercises to strengthen upper and lower limb muscle strength
	Frequency: 2 times/week	Balance exercises
		Functional exercises (The program included warm-up and recovery)
**Elliot et al. (2011)**	Duration: 20-30 minutes	Cardiorespiratory exercises-5 exercises (walking at 80% walking speed)
	Intensity: moderate to heavy (progressively increasing)	Core stabilization exercises (3 exercises)
		Upper-lower limb strengthening exercises with weights (8 reps/3 sets with progressive weight increase from 0.25 to 1.5 kg)
	Frequency: 5 times/week	Flexibility exercises and stretching (3 exercises and 4 respectively) (Total 16 different exercises)
**Mcdowell et al. (2016)**	Duration: 60 minutes	Strengthening exercises (10 repetitions upper-lower limbs-trunk and stretching exercises)
	Intensity: moderate-gradually increasing	Cardiorespiratory exercises: walking, cycle ergometer, treadmill (from 10-30 minutes and 3-4 Borg)
	Frequency: 3 times/week	(The program included warm-up and recovery)
**McWilliams et al. (2016)**	Duration: 20 minutes	Circuit Training 10 stations [(1 minute exercise/statìon)*2] (3-4 Borg)
	Intensity: moderate-progressively increasing	
	Frequency: 3 times/week	
**Vitacca et al. (2016)**	Duration: 60 minutes	Program of passive-active and assisted exercises (increasing range of motion-passive transfers-standing-stand-up/sit down)
	Intensity: progressively increasing	Breathing exercises (2 sessions daily-breathing cycle-chest expansion-violent breathing-coughing)
	Frequency: 6 times/week
		Balance exercises
		Application of electroneuromuscular stimulation to the lower limbs
		Cardiorespiratory exercises: cycle ergometer, treadmill
		Strengthening exercises
**Veldema et al. (2019)**	Duration: 20 minutes	Cardiorespiratory exercises: cycle ergometer (13 Borg Scale)
	Intensity: moderate to heavy-progressively increasing	Strength exercises with weights (4 sets/15 repetitions with a 30-40” rest)
	Frequency: 5 times/week	
**Shelly et al. (2017)**	Duration: 30-40 minutes	Ambulation
	Frequency: 5 times/week	Exercises on bed (each exercise 10times/per set)
	Ambulation twice a day (11-13 on Borg scale)	Respiratory exercises
		Dynamic quadriceps

### Outcomes and Measures

Studies have assessed functional and physical ability, muscle strength and endurance. The instruments applied were: the physical component of the SF-36 questionnaire, Barthel Index, TUG (Time Up and Go), 5STS (5-Sit to Stand), ISWT (Incremental Shuttle Walk Test), 6MWT (6 Minute Walk Test), MMS (Manual Muscle Test) and Grip Strength. All of them have often being used in terms of post ICU rehabilitation as they have been found to have significant validity and reliability in critically ill survivors [19,20].

### Functional Recovery

Functional recovery, in most studies, was assessed through the Physical component of SF-36 [10,11,13,15,17,18]. Functional ability presented significant improvements in both groups between the baseline and the completion of the intervention [10,11,17]. Between groups statistically significant differences were only noted in the study of Shelly et al after a 4-week individualized home exercise program (p=0.003). Additionally, there were studies [11,13,17] that stated that the degree of improvement was significantly higher in the intervention group. When examining general improvements over time, it could be noted that both control and intervention groups showed similar degree of improvements.

### Muscle Strength and Exercise Capacity

When assessing muscle strength, although, no study reported statistically significant difference between the groups, there were 6 studies (out of 8) that detected a higher rate of improvement in the intervention group over time in relation to the control [11,12,14–16].

Exercise capacity as usually was evaluated by 6 Minute Walking Test (6MWT) and Incremental Shuttle Walk Test (ISWT). It is mentioned that both groups were able to reach and improve beyond the minimum clinically important difference of the 6MWT and ISWT[17]. Despite that, none of the patients managed to achieve their predicted distance.

## DISCUSSION

To the best of our knowledge this is the first systematic review that focused on evaluating the functional recovery of different exercise rehabilitation programs in post ICU patients once discharged from hospital, targeting patients that continued to face functional impairments. A personalized rehabilitation program after leaving the hospital, has as its optimal goal the reduction of the functional deficits of these patients, their independence in daily activities, and finally the improvement of their health related quality of life. In order to fully describe the gains related to functionality, we additionally investigated benefits regarding muscle strength and exercise capacity. Although, improvements were found in both groups, clinically significant difference between groups was noted only in 3 studies regarding the physical component of SF-36 [10,13,18] and regarding exercise capacity in the study of McDowel et al [11]. Also, in a recent study, Veldema et al. (2019) compared the effectiveness of resistance programs and cycle ergometer training and noted improvements in walking ability and muscle strength gains, but in a short period of 4 weeks [14]. Additionally, they stated significant improvement in certain muscle groups of the lower extremity may contribute to the retrieval of both endurance and functional ability. Regarding muscle strength, Vitacca et al. [16] manifested significant improvement in respiratory muscles, as well, underlining another key component which most of the times is not assessed. Improvements over time in muscle strength that noted in the majority of studies could be strongly related to the beneficial effect that is manifested in functional ability, as these two are closely connected [20,21]. We should not neglect small beneficial effects as those seen in components like endurance as it is believed to accelerate the natural recovery process in the short term [15]. Thus, rehabilitation programs are successful in accelerating the physical recovery process in a short term period, and we fail to notice significant differences between groups at the end of the program [8,15]. The rate of recovery should be taken into consideration when designing an intervention, in order to deliver each component at the optimal time point of recovery. This describes the need for an immediate referral of critical survivors to rehabilitation facilities and a careful progression of the intervention. There was a significant difference among studies and even among the patients of the same study regarding their time of enrollment. Let alone at what time point they were evaluated for inclusion. Additionally, we should underline the fact that in a few studied when MCIDs where taken into consideration, these were surpassed [10,11].

Compliance and adherence of patients are key elements to secure the successful implementation of an intervention but is not often assessed. Unsupervised sessions raise questions on the adherence of the participants and the degree of patients’ effort. Positive effects seen in the beginning of the program, which are thought to enhance natural recovery were described by Batterham et al. (2014) and Mc Dowel et al (2017), but not by Elliot et al (2011). The explanation for this difference might had to do with the increased compliance of the intervention group participants. In unsupervised sessions, it is difficult to assess whether the patients exercise at the intensity they were instructed. The presence of supervision by trained physiotherapists reduces the chances of any errors by the participants and enhances their performance [11]. In addition, it is worth underlining that special attention is required in the education of patients to carry out an individualized rehabilitation as a few of them could have cognitive limitations [17]. These were also underlined in a previous integrative review, and added the need of a detailed educational package along with wearable devices that could monitor performance [22].

In General, the critically ill survivors are a quite heterogenic population (age, comorbidities, premorbid status), thus creating serious obstacles in interpreting the results from different studies [23,24]. Between the included studies there is a variance in age: from 40 years up to 68 years old and in relation to the admission aitiology (respiratory was stated in a higher percentage in 4 studies). The difference in the enrolled populations is also a vital component in the difficulties that we faced when interpreting the different results among the studies. And could be a reason for not being able to detect significant difference among groups at the end of the trials. Patients with a longer ICU and hospital stay and even with a prolonged period under mechanical support could present delayed functional recovery [11]. Mc Williams et al. (2016) did perform a subgroup analysis taking into consideration the length of mechanical ventilation and demonstrated greater improvements in the subgroup of patients ventilated >14 days. Connolly et al. (2015) noted that patients with ICUaw were able to achieve the same degree of functional recovery with patients without ICUaw through a tailored rehabilitation program. It is worth noting that quite a few studies were under powered, thus without the appropriate population in order to detect changes among groups [10,14,15,17,18]. Often is discussed the use of a core of outcome measures set in order to be able to create more homogenous trials and better compare and interpret results from different RCT’s. Additionally, we should look into when an outcome is self-reported or it is measured by a valid instrument, as differences could be related to patients’ perception. Different results were noted between the studies that used the physical component of SF-36 [10,11,13,15,17,18] and other instruments such as TUAG test [14,17], with the exception of Connoly et al. and Shelly et al. It is recommended that both should be used with ICU patients [19].

The rehabilitation programs either were based on strength training or were based on the principles of pulmonary rehabilitation, not always being most appropriate for the patients that were being enrolled. Critically ill survivors often present cardiorespiratory and muscular deconditioning, at a severity that changes through the recovery pathway. The trajectory of recovery could be different and unique and certainly not linear [25]. The role of cardiopulmonary exercise testing (CPET) had been discussed as a more practical method to detect which patient may benefit most [26] and to better prescribe the rehabilitation program tailored to the special needs of its patient. CPET was found to be safe for critical ill patients and underlined the presence of reduced exercise capacity in patients that were being under mechanical ventilation for >14 days. The impaired muscle oxygen utilization caused by mitochondrial myopathies could be linked to a long term impaired exercise capacity [27]. Selecting the most appropriate outcome measure could be itself challenging and must be linked to the nature of the intervention and its goal [23,24,28]. In relation to functional ability, which is the main topic of this systematic review, just using a questionnaire or just one instrument may not be enough. Functional activities may require strength, flexibility, postural control, endurance, cognitive processing etc [10, 29]. As, recovery is not linear, it is well stated that is of high importance to re-organize rehabilitational interventions in order to continuously offer the appropriate stimulus in order to maximize recovery effects. This most be done in all the components mentioned above of the rehabilitation program. In most of the studies s modified Borg scores and percentage of heart rate were used to monitor exercise intensity, and for safety purpose in unsupervised sessions, but no information were given in relation to the progression of the program. Only Elliot et al have given a more detailed description. Another point that should be consider in future studies is the evaluation or even the prescription of nutritional support as, it plays a vital role to physical recovery [30]. None of the studies included provided data in relation to nutrition. Only Connoly et al [17] have included information regarding the importance of nutrition in an educational package. Since optimization of diet and nutritional status translates into improved function, cognition and mental health nutrition should be considered an essential component to ICU rehabilitation and recovery [31]. It is well stated by Merriweather and Walsh that nutritional care of critical illness survivors is problematic and strategies to overcome these issues need to be addressed in order to improve nutritional intake [32]. As muscle wasting and weakness are major contributors to functional impairment and not only, it is of high importance to incorporate lean body mass assessment in order to predict metabolic reserve and optimize nutritional support. Non-invasive bedside measurements such as ultrasound could offer us a clear pathway of the patients’ trajectory from the acute to catabolic phase and to recovery phase when nutritional delivery and anabolic agents are more needed[33].

The aim of this systematic review was to focus on functional rehabilitation of critical ill survivors after hospital discharge. As, it is noted that decline in functional ability after ICU has been associated with ICU readmission[34]. It is important to underline the fact that only a small number of RCTs were identified for inclusion. So, this is a limiting factor for reaching clear conclusions.

All, these encourage further research on the topic. We need new randomized controlled trials with a larger sample that include the simultaneous assessment of psychological and physical variables of patients discharged from the ICU, with valid and reliable tools. Then, functional rehabilitation programs should be implemented for a longer period of time and there should be at least one re-evaluation to examine the maintenance of results over time. Furthermore, further research is needed to examine the effect of cardiorespiratory exercises compared to the program of resistance exercises, as the research included in the present work was of short duration and involved a small sample of patients, to strengthen the results. The available relevant literature studying functional capacity is not enough to produce a reliable result. More research is needed on the timing of initiation of specialized programs, as well as the intensity of exercises to achieve optimal outcomes for survivors discharged from Hospital.

## CONCLUSION

Rehabilitation of survivors of critical illness remains of high significance for patients, their families, and the health system itself. The review’s rehabilitation programs were shown to be beneficial in terms of improving features of endurance and muscle strength and functionality. However, there was no statistically significant difference between the groups. Key issues regarding recovery of critically ill patients that should be implemented not only in research but in clinical practice are the use of innovative technologies to early identify patients that will have a prolonged rehabilitation and to provide vital information regarding the status and the nutritional need of muscle mass. Rehabilitation should be tailored and continuously evolve in terms of intensity and diversity. Nutritional support should not be neglected as it has adequate importance as exercise does. Therefore, it is deemed necessary to carry out new randomized experimental studies, in order to highlight positive results for the benefit of patients. In this particular topic, there is much room for future research in order to optimize post ICU rehabilitation program from with the ICU till patient’s return to community.
